# The secretome as a biomarker and functional agent in heart failure

**DOI:** 10.20517/jca.2023.15

**Published:** 2023-06-09

**Authors:** Obed O. Nyarko, Carmen C. Sucharov

**Affiliations:** 1 Integrative Physiology Graduate Program, University of Colorado Anschutz Medical Campus, Aurora, CO 80045, USA.; 2 Department of Medicine, Division of Cardiology, University of Colorado Anschutz Medical Campus, Aurora, CO 80045, USA.

**Keywords:** Secretome, circulating protein, circulating microRNA, heart failure, biomarker

## Abstract

Heart failure (HF) is a complex and multifactorial disease. Recent advances have been made in understanding the underlying molecular processes involved in HF pathogenesis. These scientific advancements have brought to light the importance of the secretome. This paper presents a thorough overview of the state of science regarding the secretome's involvement in the onset, progression, and possibility of improved diagnosis and therapeutic interventions in HF. We explore the various types of secreted factors, including novel proteins, growth factors, cytokines, and microRNAs. We also discuss how they affect cellular signaling, angiogenesis, fibrosis, pathological cardiac remodeling, and inflammation in HF. Furthermore, we examine the role of the secretome in cardioprotection and cardiotoxicity. This review emphasizes the potential of the secretome for biomarker discovery. This might enable better HF diagnosis, risk stratification, monitoring and treatment. The review also discusses the difficulties on investigating the role of secreted factors and novel directions on secretome research. It highlights its potential as a target for novel therapeutic approaches and biomarker development.

## Graphical Abstract

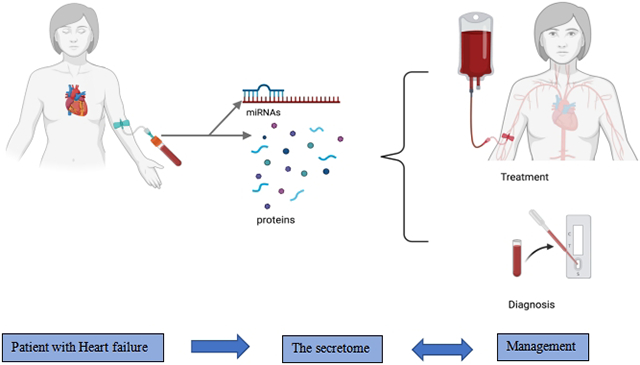

## INTRODUCTION

Cardiovascular disease remains the leading cause of death globally and includes a large group of conditions such as hypertension, coronary artery disease, and heart failure^[1]^. A diagnosis of heart failure is devastating, not only for the health of the individual, but also due to the high costs to the health system. Furthermore, the global prevalence of heart failure is projected to rise globally^[2]^. Heart failure is a complex chronic progressive disease where the heart cannot pump enough blood to meet the body’s needs^[3]^. Clinically, heart failure is a heterogenous disease that can result from a range of congenital to acquired medical conditions. Patients with heart failure usually present with difficulty breathing, cough, fluid retention, reduced exercise tolerance or may remain symptomless in the early stages of the disease. In the past few decades, there have been advanced medical and pharmaceutical therapies that have played a key role in reducing the burden of heart failure. However, heart failure still negatively impacts millions worldwide. This calls for better diagnostic and therapeutic options in the management of heart failure, which can be further improved by exploring novel molecules and how they influence cardiac health.

Biological markers can serve as indicators of disease progression or response to treatment. Presently, the commonly used biomarkers for assessing cardiovascular health include lipid profile, B-type Natriuretic Peptide (BNP), C-reactive protein (CRP), cardiac troponins, creatinine kinase, serum glucose and fibronectin^[4]^. None of these markers has proven ideal to determine disease progression. New studies have explored the secretome as a potential biological milieu of factors that can aid in the diagnosis and treatment of heart failure. The secretome consists of molecules actively secreted into the extracellular space^[5]^. This includes microvesicles, exosomes, growth factors, cytokines, chemokines, proteases, autocrine and paracrine hormones, coagulation factors and constituents of the secretory pathway^[5-7]^. The human secretome plays a significant role in cell communication, cell signaling and cell migration^[7,8]^. It can be utilized as diagnostic and/or therapeutic target^[5]^. The secretome may also exert protective or detrimental effects on the heart. Some secreted molecules have been extensively reported in literature while others have received little to no attention from the scientific community. Importantly, studies have shown that secreted molecules, such as fibroblast growth factor 21 (FGF21) and growth differentiation factors (GDFs), can impact cardiac pathologic remodeling^[9,10]^.

## PATHOLOGIC REMODELING

Pathologic remodeling of the heart is characterized by morphological and molecular changes^[11]^. Morphological changes are exemplified by changes in heart size, cavity diameter, appearance of scarred areas after a myocardial infarction (MI), fibrosis, wall thickness and inflammatory infiltrates. These changes can, initially, be compensatory, but eventually progress to maladaptive decompensation. Molecular changes include the re-expression of the fetal gene program (FGP) and a shift in metabolic substrate utilization. Activation of the FGP is an important aspect of pathologic remodeling, and one of the mechanisms the failing heart adapts to pathological insults. Activation of the FGP is widely used as a hallmark of pathological remodeling and heart failure in mammalian studies^[12,13]^. It is characterized by a reversal of gene expression from an adult profile to a fetal expression profile. The genes usually associated with the FGP include atrial and brain natriuretic peptide (ANP and BNP), α and β myosin heavy chain (MHC) and sarcoplasmic/endoplasmic reticulum Ca^2+^ ATPase 2a (SERCA2a)^[13,14]^. Upregulation of ANP, BNP, and β-MHC as well as down regulation of α-MHC and SERCA2a are associated with activation of the FGP in mammals^[12]^. As SERCA2a regulates cardiomyocyte contraction and re-uptake of calcium ions into the sarcoplasmic reticulum^[14]^, and α-MHC is associated with increased contractile force and velocity in the heart, re-expression of the FGP likely has important functional consequences to cardiac function^[12,15]^. This switch is accompanied by a switch on substrate utilization from using fatty acids as the predominant energy substrate to the use of carbohydrates as the predominant energy source in a failing heart^[16,17]^. Molecular and morphological remodeling is associated with functional changes, including cardiac dysfunction and arrhythmias. Therefore, understanding the molecular mechanisms that promote pathologic remodeling are important to identify novel therapeutic targets. In this review, we will evaluate the current knowledge related to secreted molecules altered in heart failure, and discuss their potential role in pathologic remodeling and/or cardiac function, as well as their potential as disease biomarkers.

## ROLE OF THE SECRETOME IN HEART FAILURE

The secretome plays a crucial role in the proper functioning of the cardiovascular system through the action of secreted molecules. They are vital in cardiac growth, cell to cell communication and are crucial in the cardiac pathological process^[18]^. The secretome refers to proteins, microRNAs and vesicles secreted by the heart or other organs. In recent years there has been an increase in our understanding of the secretome and its role in health and disease. The secretome is vital in disease states as it can be a target of therapeutic interventions and also prove useful as a biomarker in the diagnosis of diseases. Secreted proteins include cardiokines, which are generally secreted by cardiomyocytes and fibroblasts. These cardiokines are released under a wide range of cardiac conditions including but not limited to ischemic-reperfusion injury, oxidative stress, myocardial infarction and cardiac fibrosis^[19]^. Cardiokines of interest that have been reported in the literature include FGF21, GDFs, BNP, ANP, High Mobility Box Group Box 1 (HMGB1), C-C motif chemokine, Secreted frizzled-related protein 1 (SFRP1), Tumor Necrosis Factor-alpha (TNF-α), and Vascular Endothelial Growth Factor (VEGF). Proteins can be secreted from the heart in response to stress, as an adaptive mechanism or as a means to communicate with other organs. microRNAs (miRNAs) are short noncoding RNAs that are 18-22 nucleotides long^[20]^. Extracellular vesicles (EVs) are created by cells and have a crucial role in disease development. They also function as biomarkers because they carry miRNAs^[21]^. The function of circulating miRNA is generally accomplished through EV-mediated intercellular transfer, which protects and sorts miRNAs^[22]^. Mammalian cells release various types of EVs containing miRNAs that can be transferred to recipient cells to affect mRNA stability and cell transcriptome^[21]^.

The secretome plays an essential role in regulating diverse physiological effects on cardiac health. The secretome can be protective or detrimental to the human heart under various conditions. It can also be an essential component of basic and clinical research by serving as biomarkers to predict clinical outcomes. In this review, we explore secreted molecules that play a key role in the diagnosis, prognosis and management of heart failure in both children and adults. Secreted molecules that may be useful as biomarkers include natriuretic peptides, C-reactive proteins, cytokines and microRNAs. Secreted molecules that can play a key role in exerting biological effects include FGF21, GDFs, Midkine, HMGB1, sFRPs, endothelin, siglecs, thrombospondins, NM23, cytokines and miRNAs.

## SECRETED PROTEINS

Secreted proteins account for about a fifth of the entire human proteome and are actively transported out of the cell^[5]^. The human body consists of numerous cells that secrete proteins^[23]^. Although the main organs in the body that play a key role in protein secretion are the bone marrow and liver, there are numerous proteins produced and secreted by cardiomyocytes that have been implicated in heart failure^[4]^. In fact, the heart produces and releases proteins, also called cardiokines. A cardiokine is defined as a peptide or protein that is present in the bloodstream and signals to distant tissues in an endocrine manner^[24]^. It has a tissuespecific expression pattern and is secreted by the heart. It exists in a free form and directly interacts with ligand receptors, rather than being a part of extracellular vesicles^[24]^. In disease, these proteins are probably released as a result of disruption to the heart’s metabolic process or as an adaptive mechanism to the reduced cardiac output. Multiple cell types in the heart such as cardiomyocytes, fibroblasts and endothelial cells are implicated in the secretion of proteins from the failing heart. These cells have also been shown to secrete cardiokines under stressful conditions such as ischemic/reperfusion injuries. These secreted proteins may act in a paracrine or autocrine manner and may be difficult to quantify and access systematically^[7]^. Some secreted proteins such as cytokines may also act in an endocrine manner^[4]^.

## THE HUMAN PROTEOME IN HEART FAILURE

In this section we will discuss the current knowledge of secreted proteins and their potential role in pathologic remodeling. These are summarized in Table 1.

### Natriuretic peptides

The heart not only serves as the primary organ of circulation, it also has an endocrine function. Natriuretic peptides are protein hormones that are classified as such primarily because they are involved in natriuresis. Natriuretic peptides play a role in maintaining the compensated state in the early stages of heart failure and in inhibiting the renin-angiotensin-aldosterone system. The heart increases the production and secretion of natriuretic peptides in response to volume and pressure overload in heart failure. As such, they have emerged as important biomarkers in the diagnosis and prognosis of acute and chronic heart failure.

### Brain natriuretic peptide

Brain natriuretic peptide (BNP) is so named because it was initially sequenced from the brain of pig tissue^[25]^. It was subsequently discovered in higher quantities in cardiac tissue and is mainly secreted by the left ventricle^[26]^. BNP also plays key roles in the inhibition of the renin-angiotensin-aldosterone system, decreases vascular pressure, and promotes angiogenesis, inhibits cardiomyocyte hypertrophy and apoptosis amongst others^[27]^. BNP is secreted in cases of stressful cardiac events such as volume overload. Plasma concentrations of BNP are increased in conditions such as congestive heart failure^[28]^ and is hence used as reliable biomarkers in the diagnosis of heart failure.

BNP is a 32 amino acid protein initially synthesized as pre-pro BNP, a 134 amino acid peptide^[29]^. Its N-terminal signal peptide is cleaved resulting in the mature/active peptide and an inactive fragment called NT-proBNP (N-terminal pro-BNP)^[30]^. Both the pre-prohormone and mature BNP are found in the circulation^[27]^. In heart failure patients, there is a relatively lower level of mature BNP but higher levels of the pre-prohormone^[27,31]^. BNP has been shown to act locally to reduce fibrosis of the ventricles^[31]^. BNP interacts with the guanylyl cyclase/natriuretic receptor A to produce downstream effects via the second messenger cyclic guanylyl-monophosphate (cGMP)^[27]^. Cardiomyocyte restricted deletion of guanylyl cyclase A in mice leads to mild cardiac hypertrophy and the expression of markers of hypertrophy^[32]^. Only the mature BNP interacts with the receptor to produce downstream effects. While NT-proBNP is used as a diagnostic marker for heart failure, it does not have biological activity and does not exert any physiological effects^[29]^. Because of the differential plasma concentration of the pre-prohormone and mature BNP, there are various possible utilities of BNP both as a biomarker and therapeutic target in heart failure, coronary artery disease and myocardial infarction^[33]^. In recent years, the signal peptide of BNP (BNPsp) has also been explored for use as a biomarker for cardiac ischemia prior to necrosis^[34]^. Elevated BNP has been associated with increased mortality and risk of heart failure^[35,36]^. Genetic variants of BNP have varied phenotypic expression and effects on cardiometabolic risk factors. These important clinical phenotypes associated with genetic functional variants can further enhance the current diagnostic and therapeutic relevance of natriuretic peptides^[36]^. A functional variant in the promoter region of BNP gene (NPPB), the rs198389 has been is associated with elevated levels of NT-proBNP^[35]^. The functional variant is also associated with improvement in overall cardiovascular mortality, hypertension and increased life expectancy^[35]^. The BNPsp is the 26 amino acid N-terminal end of the pre-proBNP that is cleaved during maturation^[27]^. The signal peptide of BNP exists as a distinct molecule also found in circulation and useful as a biomarker of heart disease specifically myocardial infarction and cardiac ischemia^[34]^. Interestingly, BNPsp was not found in the circulation of patients with heart failure, differently than pre-proBNP and mature BNP^[34]^. This suggests the different forms of BNP are associated with specific cardiac-related diseases.

### Atrial natriuretic peptide

Atrial Natriuretic Peptide (ANP) is highly evolutionary conserved across species^[37]^. ANP is released by cardiac tissue in response to atrial distention/stretch^[38]^. It primarily serves to inhibit the activation of the renin-aldosterone system and regulate sodium homeostasis ^[38]^ Two known receptors interact with ANP, the ANP receptor A (ANPR-A) and the clearance receptor C. The ANP receptor A activates adenylyl cyclase to produce 3′5′-cyclic monophosphate (cGMP) and effect biological changes mediated by ANP^[31]^. The clearance receptor C of ANP is the most abundant and is responsible for clearing ANP from circulation. ANP also has a biological inactive prohormone called the pro-atrial natriuretic peptide (N-ANP) which is found in circulation together with the biological active C-ANP^[31]^. The prohormone is relatively more stable and is found in higher concentrations than the biologically active ANP. The plasma half-life of ANP has been reported to be within the range of 1.7 and 3.1 min. It is found in relatively low concentrations in healthy humans but increases 10 to 30-fold in patients with congestive heart failure^[38]^.

### Fibroblast growth factor 21

Fibroblast growth factor 21 (FGF21) is a relatively novel polypeptide ligand that has been shown to exert cardioprotective effects in various forms of heart diseases^[39]^. FGF21 is part of the family of fibroblast growth factors involved in a plethora of pathophysiological processes ^[40]^. It is a metabolic hormone that has paracrine, autocrine and endocrine functions involved in the regulation of glucose and lipid metabolism^[39]^. FGF21, through interaction with FGF receptors, mediates fasting glucose response by increasing insulin sensitivity, increasing glucose uptake and reducing serum glucagon and triglyceride levels^[40]^. FGF-21 is mainly secreted by the liver^[41]^ and adipose tissue^[42]^ but can also be produced by cardiac^[43]^ and skeletal muscle cells^[43]^. In addition to its metabolic effects, FGF21 has also been shown to have cardioprotective effects through interaction with the FGFR1c and reversal of maladaptive hypertrophy^[44]^. FGF-21 also protects against ischemic/reperfusion injury in rat hearts by reducing caspase 3 activity and cell death^[45]^. The liver and adipose tissue increase their production and release of FGF21 in response to myocardial injury, which helps protect the heart through phosphorylation of the FGFR1/β-Klotho-PI3K-Akt1-BAD signaling network, reducing caspase 3 activity^[46]^.

Studies have shown that FGF21 levels are associated with risk prediction and can aid in determining the prognosis of patients with cardiovascular diseases^[47]^. Relatively high baseline levels of FGF21 are associated with adverse outcomes in patients with acute heart failure (AHF). Elevated FGF21 levels at the start of treatment of AHF are linked to negative clinical outcomes^[48]^. Serum circulating FGF-21 have also been found to be elevated in patients with heart failure with reduced ejection fraction and to correlate with BNP levels^[49]^. This suggests that measuring serum FGF21 levels could be a useful biomarker for predicting outcomes in patients with heart failure.

### Growth differentiation factors

The growth differentiation factors (GDFs) are a group of proteins that belong to the transforming growth factor-beta (TGF-β) superfamily^[50]^. The GDFs play important roles in cell differentiation, growth, and development in various tissues and organs, including the heart^[51]^. GDFs exert varied and sometimes opposing effects under different pathological conditions by regulating apoptosis, angiogenesis, and inflammatory response^[52]^. GDF-15 is the main GDF in the setting of cardiovascular disease. GDF-15 secretion occurs when the body experiences oxidative stress, inflammation, or hypoxia^[53]^. In the heart, GDFs are involved in the regulation of various cellular processes, such as cardiac development, cardiomyocyte proliferation, and cardiac remodeling in response to injury.

GDF-15 is a cardiac hormone predominantly induced by injury and may be a useful biomarker in the diagnosis of cardiac diseases^[51]^. GDF-15 is not only important in heart disease but also serves as an inflammatory marker in other ailments such as cancers, neurodegenerative and ischemic diseases^[54]^. GDF-15 is also associated with major adverse cardiovascular events in patients with coronary artery disease^[55]^. GDF-15 plays a key role in the development and progression of cardiovascular diseases. Serum circulating levels of GDF-15 can reflect the inflammatory process in atherosclerosis and coronary artery disease^[52]^. GDFs such as GDF-15 can be explored as a novel biomarker in the diagnosis of various heart conditions such as acute coronary syndrome^[52]^. GDF-15 is also known to be a marker of mitochondrial dysfunction, and it can regulate appetite and energy expenditure in response to metabolic stress^[56]^.

Circulating GDF-11 has been discovered as an anti-ageing factor in age-related cardiac hypertrophy in mice^[57]^. GDF-11 exerts antihypertrophic effects in young mice and its levels are decreased with increasing age^[57]^. GDF-11 has also been shown to protect against ischemia/reperfusion injury, promote cardiac regeneration by enhancing cardiomyocyte proliferation and reducing fibrosis in the injured heart^[58]^. Higher levels of GDF-11 have also been associated with reducing the risk of adverse cardiovascular events supporting the protective roles of GDF-11on the heart^[59]^. GDFs play important roles in the regulation of cardiovascular function and response to cardiac injury. Systemic delivery of these molecules could potentially treat multiple cardiovascular diseases. Nonetheless, further research is necessary to fully comprehend GDF mechanisms in the heart and to identify the most effective strategies to target these molecules for therapeutic advantages.

### Myosin heavy chain proteins

Myosin-heavy chain proteins are motor contractile proteins that drive the heart's contractility via ATP hydrolysis. Cardiac thick filaments have myosin-heavy chains as their main motor proteins^[60]^. Each chain is made up of a globular head connected via an elastic hinge to a long tail^[61]^. In the heart, α-MHC and β-MHC are the primarily expressed myosin isoforms and are found on chromosome 14^[62]^. These 2 isoforms exist in the ventricular myocardium and their relative expression is associated with the contractile power of the heart^[62]^. α-MHC protein has been shown to have a relatively higher ATPase activity compared to β-MHC. Under conditions of stress, the heart undergoes numerous changes not only phenotypically, but also at the cellular and molecular levels. Changes in gene expression can lead to the secretion of proteins that are elevated in pathological cardiac remodeling. Arguably, one of the most significant changes that occurs in cardiac pathological remodeling is the differential expression of the α-MHC and β-MHC genes. Small mammalian hearts have higher contractile force and velocity which is associated with higher levels of α-MHC^[62]^. In humans, studies have shown that although levels of α-MHC are low, they are decreased in disease^[62]^. Cumulative circulating serum MHC levels are significantly associated with the cumulative release of creatine kinase (CK), lactate dehydrogenase (LDH) and Creatine Kinase-MB (CK-MB) in different patient groups^[63]^. Elevated CK, CK-MB and LDH are often indicative of acute myocardial infarction. A study in dogs indicated that circulating myosin could be useful as an indicator of cardiac muscle necrosis^[63]^. Comparatively, the measurement of fragments of serum myosin heavy chain from skeletal muscle has proven useful in the diagnosis of rhabdomyolysis^[64]^. The levels of MHC protein can not only be used as a marker of heart muscle disease (necrosis) but can also be exploited in developing appropriate therapies in the management of cardiomyopathies. In fact, the COSMIC-HF trial, using the cardiac myosin activator omecamtiv mercabil showed promising results^[65]^.

### C-reactive protein

C-reactive protein is the most commonly used acute phase reactant in the diagnosis and prognosis of heart disease. Circulating CRP is a plasma protein found in higher concentrations in the acute phase of an inflammatory cascade^[66]^. CRP is mainly produced by the liver in humans; however, it is also produced by smooth muscle cells^[67]^, adipose tissues^[68]^ and macrophages^[69]^. Higher levels of CRP are associated with inflammation^[66]^. CRP is a major component of the innate immune system and induces apoptosis in the vascular endothelium and causes vascular endothelial cell dysfunction^[70]^. It was also shown to decrease endothelial nitric oxide synthase and to play a key role in vascular remodeling in the pathogenesis of atherosclerosis^[70]^. CRP is a biomarker of cardiovascular diseases including vasculopathy and cardiomyopathy^[71]^. Elevated circulating CRP is a predictor of adverse outcomes particularly in patients with acute coronary syndrome at risk of severe complications^[72]^. CRP is found in relatively higher concentrations in the myocardium of patients with cardiovascular diseases and is associated with microvessel rarefaction, endothelial cell toxicity and cell apoptosis^[73]^. Antioxidants have been found to reduce CRP toxicity in the myocardium^[71]^. CRP is well recognized as a marker of acute infection and has also been used as a marker and prognostic indicator of heart disease. CRP has been shown to promote atherogenesis making it deleterious in heart diseases^[67]^. CRP is highly sensitive but not very specific for cardiovascular diseases^[67]^. This limits its use as an ideal biomarker in patients with heart failure.

### Troponins

Clinically, the diagnosis of myocardial infarction relies heavily on the use of cardiac troponins. Cardiac troponin T and I are regulatory proteins highly elevated in patients with myocardial infarction^[74]^. They are highly specific to cardiac tissue, and genes coding for troponins are unique to the myocardium^[74]^. Cardiac troponins play a regulatory role in the contraction of cardiac muscle. Troponins are thin filament proteins^[75]^. Troponin C is an 18kDa amino acid protein that binds calcium ions in the contractile apparatus. Troponin I is a 27 kDa amino acid protein that serves as the inhibitory component of the actomyosin crossbridge formation^[76]^. Post-translational modifications (phosphorylation) of troponins regulate the troponin-tropomyosin complex and influence cardiac contraction^[75]^. Troponins are found in very low concentrations in the serum of healthy patients. Cardiac troponins are also found to be persistently elevated in patients with cardiomyopathy and heart failure and are indicative of myocardial damage^[77]^.

## NOVEL PROTEINS

In addition to the well-established secreted proteins implicated in the pathogenesis of heart failure, numerous novel proteins are currently being explored for their roles in cardiac growth and pathological remodeling. Numerous factors in the secretome affect the changes that precede and/or occur during pathological remodeling. New molecules are being explored for their roles in cardiac remodeling and contractile dysfunction in decompensated heart failure^[62]^.

### High-mobility group box 1

High-mobility group box 1 (HMGB1) is ubiquitous and plays different roles in mammalian systems depending on its cellular location. HMGB1 is a non-histone DNA-binding protein that regulates transcription and autophagy, and plays a role in the repair of DNA^[78]^. HMGB1 is made up of 215 amino acids organized in the Box A and B domains^[78]^. The different A and B domains have contrasting features. Box A is anti-inflammatory while Box B is pro-inflammatory. HMGB1 is involved in myocardial necrosis and inflammation, which are key contributors of cardiac remodeling and activation of the FGP. Both extracellular and cytoplasmic HMGB1 play a vital role in cardiac remodeling^[79]^. A study showed that neonatal rat cardiomyocytes (NRCM) had increased expression of HMGB1 in both the intracellular and extracellular spaces when subjected to mechanical forces^[80]^. HMGB1 is also a danger-associated molecular pattern (DAMP) secreted by necrotic cells. It activates the innate immune system to induce inflammation in cardiovascular diseases^[79,81]^. Due to its abundance in eukaryotes, it has been well-studied and shown to have varied biological effects. In the cardiovascular system, it is debated if its role is protective or deleterious. This has made the development of therapeutics to target HMGB1 very challenging. HMGB1 plays a key role in cardiomyocytes by regulating cardiac function via metabolic cellular changes. In cardiomyocytes, HMGB1 is found in the nucleus and in the cytoplasm^[81]^. Under stressful and ischemic cardiac conditions, nuclear HMGB1 translocates into the cytoplasm and can be exported into the circulation stimulating the secretion of pro-inflammatory cytokines, enhancing macrophage migration and activating pro-inflammatory receptors. Activation of certain receptors, such as Toll-like receptors, leads to inflammation^[81]^. This inflammatory cascade leads to cardiac dysfunction, remodeling, hypertrophy and eventually cardiomyopathy and heart failure. Elevated circulating HMGB1 is associated with myocardial infarction in humans. In rodent myocardial infarction models, HMGB1 plays a significant role in the pathological remodeling of the left ventricle^[82]^. In sharp contrast, HMGB1 has also been shown to maintain normal cardiac function in mice^[83]^. Deletion of cardiac-specific HMGB1 leads to left ventricular dysfunction and impairs the growth of the heart^[83]^. This study by Peng and colleagues demonstrated the key role HMGB1 plays in cardiomyopathy and heart failure^[83]^. In experimental models of cardiomyopathies induced by various stimulants, the inhibition of extracellular HMGB1 was found to be cardioprotective and reduce inflammation^[84]^. Interestingly, mice with over-expression of cardiomyocyte-specific HMGB1 have some form of cardioprotection^[84]^. Another study also found that generally higher levels of circulating HMGB1 are associated with cardiac myopathies^[85]^. HMGB1 is a protein of interest because it elicits diverse and, in some cases, contrasting physiological and pathological effects in the human adult heart^[86]^. HMGB1 is critical to the development of heart failure, and studies in human patients will be crucial to define its exact role in cardiomyocytes and pathological remodeling and how that can be exploited in the management of heart failure.

### Secreted frizzled-related proteins

Secreted frizzled-related proteins (sFRPs) modulate the WNT signaling pathway^[87]^. sFRP-1 was discovered as the first antagonist of WNT signaling. This pathway is highly conserved and crucial in cellular differentiation, proliferation, death and early embryonic development^[88]^. Components of the WNT signaling pathway have long since been explored as targets for therapeutics in both cancers and cardiometabolic diseases^[89]^. WNT signaling can happen through a β-catenin-dependent (canonical) and independent cascade (non-canonical). Both the β-catenin-dependent and independent pathways have been implicated in vascular smooth muscle cell (VSMC) remodeling (such as SMC proliferation. apoptosis and migration), in atherosclerosis, cardiac hypertrophy and other cardiometabolic diseases^[90,91]^.The aberrant canonical WNT pathway has long been linked to tumor growth^[90]^. sFRPs bind to extracellular WNT ligands and frizzled receptors^[91]^, and this interaction has been shown to suppress tumor growth. sFRPs were hence initially classified as tumor suppressors, even though they have also been shown to cause cell growth under some conditions^[90]^. There are five known sFRPs (sFRP1-5) and they are known to regulate cellular apoptosis, differentiation, and angiogenesis and play a role in the inflammatory cascade^[45]^. sFRPs are expressed in different tissues including the heart. sFRP-1, sFRP-2 and sFRP-5 are expressed in the heart and play a role in cardiac development and function^[91]^. This review will focus on sFRP-1 due to its potential therapeutic significance and role in age-related cardiac deterioration. Although the bulk of this review will focus on the effect of sFRP-1 in cardiomyocytes, sFRP-1 is also important in fibroblast homeostasis, as cardiac fibroblast lacking endogenous sFRP-1 potentially predisposes cells to cardiomyopathy during aging^[92]^.

There are different reports in the literature on the effects of sFRPs on cardiomyocytes. sFRPs may promote inflammation, collagen deposition, and fibrosis in healing cardiac tissue^[91]^. Earlier research has demonstrated that sFRPs have varying effects on myocardial fibrosis^[93]^, hypertrophy^[94]^, angiogenesis^[95]^, cardiac cell death^[96]^, and regeneration^[97]^. This occurs through either activating the non-canonical WNT pathway, modulating other signaling pathways, or bi-directionally regulating the canonical WNT pathway. sFRP-1 may inhibit apoptosis of cardiomyocytes by modulating the effects of glycogen synthase kinase-3β (GSK-3β) which is a down-regulator of WNT signaling^[87]^, and whose inactivation and phosphorylation is cardioprotective^[98]^. Over-expression of sFRP-1 has been shown to reduce the size of cardiac infarcts and improve cardiac function. The cardioprotective role of sFRP-1 has been further suggested by other studies, which showed that sFRP-1 improves cardiac dysfunction by inhibiting cardiomyocyte apoptosis induced by the WNT signaling pathway^[90]^. This cardioprotective role of sFRP-1 may be exploited as a key target in the development of therapeutics to manage heart disease^[45]^. Interestingly, other studies showed that overexpression of sFRP-1 impairs phosphorylation and activation of GSK-3β and inhibits cardioprotection after ischemic preconditioning in an *in vivo* model^[99]^. Furthermore, our group showed that increased serum circulating levels of sFRP-1 can also promote cardiomyocyte stiffness^[13]^.

sFRP-1 has a biphasic effect on WNT signaling; increasing WNT activity at low concentration and reducing WNT activity at high concentrations^[87]^. sFRP-1 can inhibit WNT signaling in cardiomyocyte development^[100]^. Modulating WNT signaling might be an important factor in the reactivation of the FGP because WNT proteins play a crucial role in cell development and regeneration^[89]^. sFRP-1 overexpression is associated with an increased risk of cardiovascular diseases, coronary artery disease (CAD), diabetes and even obesity^[91]^.

As a biomarker, studies have shown that serum circulating levels of sFRPs are associated with cardiovascular diseases^[101]^. Circulating sFRPs also play a vital role in metabolic syndrome and act as a risk factor especially for patients with concomitant heart failure and type 2 diabetes mellitus^[102]^. Higher levels of sFRP-1 precede cardiovascular events and may prove a useful biomarker in cardiovascular diseases. In summary, there is ample evidence that sFRPs play a role in heart diseases and although there has been much research into it, their effects are still inconclusive. More studies are necessary to define its contribution to disease progression or improvement.

### Midkine

Midkine (MDK) was originally discovered as a heparin-binding cytokines involved in the regulation of growth and differentiation in the brain and kidney of mice^[103,104]^. MDK is a soluble secreted protein with a molecular weight of 13 kDa^[105]^. MDK plays a key role in several physiological states such as growth, differentiation and repair^[105]^. MDK is found to be highly expressed in various pathological conditions such as heart disease and cancer^[106]^. MDK promotes growth and invasion of cancers^[105]^ and can be an important cancer biomarker, aiding in the diagnosis and progression of several cancers^[107]^. MDK also plays a key role in neurogenesis and postnatal development of the hippocampus in mice^[108]^. In recent years, MDK has also been implicated in various cardiovascular diseases. However, there is still no consensus on whether MDK is beneficial or detrimental to cardiovascular diseases (extensively discussed by our group^[109]^). Our group showed that MDK can cause activation of the FGP^[13]^.

As a biomarker, serum levels of MDK are increased in patients with heart failure compared to controls^[110]^. Increased serum MDK levels have also been associated with the risk factors of atherosclerosis due to hypertension and hypercholesteremia^[111]^. Indeed, the systemic administration of MDK promotes atherosclerotic plague formation via pro-inflammatory, anti-apoptotic and angiogenic factors^[112]^. In sharp contrast, a single intracoronary injection of MDK was shown to reduce the size of an infarct and improve systolic/diastolic left ventricular dysfunction in swine hearts^[113]^. Further studies are necessary to better define the role and biomarker predictability of MDK in the various cardiovascular disease etiologies.

### Endothelin-1 and siglecs

Endothelin-1 is a 21-amino acid peptide hormone that is secreted in response to various stimuli by vascular endothelial cells^[114]^. ET-1 is mainly produced by the endothelium and cardiomyocytes^[115]^. Endothelin-1 has diverse biological functions in the human body, particularly the heart where it is associated with cardiac dysfunction^[115]^. ET-1 activates G-protein coupled receptors, endothelin type A receptors and promotes hypertrophic remodeling of cardiomyocytes^[116]^. Endothelin converting enzyme (ECE) is highly expressed in the heart and binds Sialic acid binding immunoglobulin like lectin 9 (siglec-9) to produce proteolytically active endothelin^[117]^. Persisting endothelin-1 signaling induces hypertrophic gene expression and induces pathological remodeling of the heart^[116]^.

Siglec-9 is expressed in several tissues particularly immune cells in the body^[5,118]^. They are a large family of type 1 transmembrane proteins that mediate immune signaling in both the innate immune and adaptive immune system^[119]^. Siglecs have at least two domains, an extracellular domain and a transmembrane domain^[119]^. Siglec-9 has been shown to be upregulated in the inflammatory cascade and serve as ligands for vascular adhesion molecules needed for leucocyte migration^[118]^. Siglecs can be upregulated on granulocytes and monocytes^[118]^. Most Siglecs inhibit the inflammatory response in autoimmune and inflammatory conditions^[119]^. Siglec-9 and 7 are involved in cellular apoptosis and neutrophil phagocytosis^[119]^. The intravenous administration of monocyte chemoattractant protein (MCP) and siglec-9 protects rats from acute liver injury and improves survival of rats with acute liver failure by suppressing hepatocyte apoptosis^[120]^. Siglec-9 also works synergistically with MCP to suppress inflammatory responses in cells and to promote cellular proliferation^[120]^. Siglec-9 preferentially binds to sialosides that have a sulphated epitope and an a-2,3 linked sialic acid^[121]^. Serum concentrations of sialic acids have been found to be elevated in patients with various forms of cardiovascular diseases such as coronary artery disease and atherosclerosis^[122]^. Siglec-9 is expressed on many cells in the body including B cells, natural killer cells, monocytes and neutrophils^[118,121,123]^. Neutrophils are the most important early effectors in the innate immune system. In neutrophils, Siglec-9 induces both apoptotic and non-apoptotic death pathways^[124]^. Siglec-9 is proinflammatory, and antibodies developed against siglec-9 are part of the active components of intravenous immunoglobulin preparations used for the treatment of some autoimmune conditions^[125]^.The altered expression and interaction of sialic acid and siglec-9 can be explored in the development of therapeutics for heart failure. Antibodies that alter siglec-9 protein interaction may be key to mediating the inflammatory responses in heart failure

### NM23-nucleoside diphosphate kinase

Non-metastatic protein clone 23 is a nucleoside diphosphate kinase that was initially discovered as a metastatic tumor suppressor gene^[126]^. It is highly conserved in mammals and expressed in various tissues^[127]^. NM23 is responsible for maintaining the cellular equilibrium of nucleoside triphosphate by catalyzing the reversible transfer of phosphate ions between 5’ nucleoside triphosphate and 5’ nucleoside diphosphate^[126]^. NM23-Nucleoside diphosphate kinase signal transduction mechanism alters the cAMP pathway^[128]^ and circulating NM23 suppresses cAMP synthesis in patients with heart failure^[129]^. cAMP signaling is crucial in the contractile activity of the heart through the β_1_ and β_2_ adrenergic receptor pathways. Patients with chronic heart failure have lower cAMP levels due to desensitization of the β adrenergic receptor^[129]^. In cardiomyocytes, chronic activation of β adrenergic receptors increase the binding of NM23 to cardiac sarcolemma to activate G-protein coupled receptor signaling pathway. Sarcolemmal binding to NM23 induces expression of hypertrophic genes and causes cardiac hypertrophy^[130]^. Elevated levels of circulating NM23 have been found in the sarcolemmal membranes of patients with end-stage heart failure^[130]^. The specific effects of NM23 on the development and progression of heart failure is still not definite and needs to be explored further.

### Thrombospondins

Thrombospondins are matricellular proteins that play an important role in the pathology of diverse cardiovascular diseases. There are currently five known thrombospondins (1-5) all of which are expressed in the heart^[131]^. Thrombospondin 1, 2 and 4 are upregulated in pathological remodeling of the heart^[131]^. Thrombospondins have been implicated in the control of the extracellular matrix composition in hypertrophic hearts. The expression of thrombospondin 4 have been shown to be increased in hypertrophic and failing hearts^[132]^. Thrombospondin 4 has also been hypothesized to be involved in the heart’s adaptive response to stress overload and heart failure^[133]^. The extracellular matrix supports and maintains structural integrity of cardiomyocytes and aids in cell differentiation, adhesion, communication and motility^[134]^. The extracellular matrix provides architectural framework to cardiac tissues during compensatory hypertrophic remodeling. Thrombospondins increase cardiac hypertrophy by increasing extracellular matrix deposition and promoting fibrosis^[134]^. Thrombospondin 2 plays a crucial role in hypertensive hypertrophic remodeling by regulating extracellular matrix deposition^[135]^. Imbalances in extracellular matrix deposition and degradation potentially mediate cardiac fibrosis and infarction^[134]^. Rats that have an increased expression of thrombospondin 2 are relatively more prone to develop early heart failure^[135]^. Furthermore, thrombospondins are significantly elevated in patients with acute ST-segment elevation myocardial infarction^[136]^. Thrombospondins have a complex multiple domain structure that interacts with cardiac receptors, growth factors, cytokines and other matricellular proteins like periostin, osteonectin and osteopontin^[134]^. Due to its diverse interactions, it plays various roles in the pathological remodeling of the heart. Some thrombospondins might also play a protective role in patients with heart failure^[137]^. The upregulation of thrombospondin 1 in diabetic cardiomyopathy prevents cardiac dilation through the upregulation of angiopoietin-2^[138]^. Thrombospondins are closely associated with cardiac hypertrophy but its specific role in heart failure is yet to be fully investigated.

Circulating thrombospondin-2 has already been explored as a potential marker of fibrosis in nonalcoholic fatty liver disease in type 2 diabetes^[139]^. Plasma levels of thrombospondin-1 have also been shown to be elevated in patients with pulmonary hypertension^[140]^. Serum circulating levels of thrombospondin-2 have been associated with increased incidence of hypertensive heart failure and worsening diastolic function in patients with type 2 diabetes^[141]^. The expression and role of thrombospondins in the heart make them viable candidates that can further be explored as potential biomarkers or prognostic indicators in management of heart failure.

### Cytokines

Cytokines aid in cellular interactions and communication. They are small secreted proteins released by cells under various conditions^[142]^. Chemokines are cytokines with known chemoattractant effects. Cytokines may be pro-inflammatory or anti-inflammatory. Inflammation is crucial in the physiological and pathological functioning of the heart and plays a key role in post-myocardial injury repair. Chronic inflammation is, however, detrimental to the functioning of the normal heart and contributes to pathological remodeling. Inflammation can promote fibrosis, another key component of cardiac health^[143]^. Although relatively little is known about the role of specific inflammatory mediators on chronic heart disease, inflammation plays a vital role in the development of heart failure. Both the innate and adaptive immune systems are activated in heart failure and involve both cellular and non-cellular components. The immune response is initiated by the activation of danger associated molecular patterns (DAMPS) in the sterile inflammatory response or by pathogen associated molecular patterns (PAMPS) in infectious causes of heart diseases. Both PAMPS and DAMPS are recognized by the numerous toll-like receptors in cardiomyocytes, inflammatory cells and endothelial cells^[144]^. The inflammatory response is led by the infiltration of neutrophils, monocytes/macrophages and lymphocytes through endothelial adhesion molecules^[145]^. Inflammatory mediators are recruited to the site of injury in the heart via cytokines, which in cardiomyocytes is particularly due to upregulation of the chemokine CCL2^[146]^. Sustained inflammatory response however worsens heart failure. In the normal myocardium, pro-inflammatory cytokines such as IL-1β, IL-6, IL-18 and TNF-α are not constitutively expressed^[147]^. Upregulation and expression of these pro-inflammatory cytokines are detrimental to the heart in the long run. Elevated and sustained circulatory levels of TNF-α are associated with heart failure by inducing cardiomyocyte apoptosis, increasing extracellular matrix degradation and increasing oxidative stress and DNA damage^[148]^. Antagonists to TNF-α have proven effective in inflammatory conditions such as Crohn’s disease and rheumatoid arthritis, but anti-TNF-α drugs have not shown benefits in patients with chronic heart disease^[143,149]^. IL-1β effects in the heart are similar to that of TNF-α, and in addition, IL-1β increases myocardial stiffness, fibrosis and reduces β-adrenergic receptor responsiveness. The CANTOS trial using IL-1β targeted anti-inflammatory therapy with canakinumab resulted in improved outcomes in patients with myocardial infarction, with or without established heart failure, compared to placebo^[150]^. *In vivo* treatment of IL-10 in patients post-myocardial infarction improves cardiac remodeling^[151]^. Modulation of the inflammatory response in heart failure represent a key potential therapeutic target for intervention. Cardiac specific subtypes of inflammatory mediators could be particular importance as they would reduce the systemic side effects of anti-inflammatory agents.

### microRNAs in heart disease

In addition to proteins, microRNAs (miRNAs) have been highly investigated for the roles they play in the pathophysiology of heart failure. miRNAs are single-stranded and highly conserved short nucleotide sequences that post-transcriptionally regulate gene expression^[152]^. miRNAs bind to the 3′ untranslated region of mRNAs and promote the degradation or translational repression of target mRNAs^[152]^. Each miRNA targets several multiple known transcripts^[153]^ and can regulate the expression of a large number of genes that play vital roles in cellular processes such as cell growth, differentiation and apoptosis^[154,155]^. miRNAs have emerged in recent times as key players in the pathogenesis of several diseases including cardiovascular diseases. miRNAs can regulate the expression of genes that are associated with cardiac hypertrophy, fibrosis, inflammation, and apoptosis, all of which are important pathological mechanisms involved in heart disease^[156]^. Some miRNAs, such as miR-1, are muscle specific, and miR-1 influences cardiac and somatic muscle differentiation in drosophila and regulates notch signaling^[157]^. The biogenesis of miRNAs is essential to cardiogenesis as cardiac-specific deletion of miR-1-2 in mice has a profound effect in heart morphogenesis, electrical conductance and cell-cycle control^[158]^. Furthermore, studies have shown that over-expression or down-regulation of miRNAs have an important impact in cardiac homeostasis and can promote pathological remodeling in animal models^[159,160]^. In fact, one of the most abundant cardiac specific miRNAs, miR-1, is downregulated in cardiac hypertrophy in a mouse model^[161]^. Another miRNA, miR-133a also has important roles in cardiac development and disease. A previous study showed that miR-133a-1 or miR-133a-2 knockout animals were normal at birth^[162]^; however, 50% of the double knockout animals died postnatally, with only 24% of the animals surviving to adulthood. These animals displayed profound pathologic remodeling, including fibrosis, sarcomere fragmentation and disorganization that was accompanied by DCM. Interestingly however, transgenic over-expression during embryonic development (under the regulation of the β-MHC promoter) was detrimental, and resulted in cardiac failure and death, likely due to a decrease in cell proliferation^[162]^. In contrast, over-expression of miR-133a postnatally (under the regulation of the α-MHC promoter) prevented fibrosis and apoptosis in a transaortic model^[163]^. These results underscore the importance of age- and context-specific investigations.

miRNA expression in the failing human heart suggests an important role for miRNAs in the development and progression of cardiomyopathies and can impact cardiomyocyte remodeling^[164]^. These miRNAs include miR-18a, miR-21, miR-27a, miR-30e, miR-26b, miR-199a, miR-106a, miR-1228, miR-129, miR-122, miR-210-3p, miR-423-5p, miR-142-3p, miR-622, miR-652, miR-195, miR-1 and miR-92b-5p^[152, 165, 166]^. miRNAs have also been investigated as potential therapeutic targets in the management of heart failure and differential expression of myocardial miRNAs such as miR-208a-3p, miR-591, -208b-3p, 199a-5p, 21-5p and miR-1-3p is associated with reverse modeling in response to chronic β-adrenergic blocker treatment in patients with dilated cardiomyopathy^[167]^. The study of cardiac-enriched miRNAs can not only further elucidate the pathophysiology of heart disease, but can also improve upon traditional biomarkers to better the diagnosis and treatment of heart diseases

### Circulating miRNAs as biomarkers and effectors in heart disease

About 10% of miRNAs encoded in the human genome are found in the serum^[168]^. Circulating miRNAs are endogenous, stable non-coding RNAs that can be useful in the diagnosis of cardiovascular diseases. These circulating miRNAs are relatively more stable and resistant to degradation by endogenous RNase activity compared to exogenous unmodified miRNAs^[169]^. Selected miRNAs have also been detected in exosomes and in bodily fluids such as urine and saliva^[170]^. This makes circulating RNAs an interesting emerging field in disease detection. Specific circulating miRNAs have been discussed as potential biomarkers in several cancers^[171]^ and a number of pathologies^[172]^, opening up the possibility of exploring them as a minimally invasive diagnostic marker. Sensitive and specific biomarkers are vital in the early diagnosis, favorable prognosis and disease treatment.

In the cardiovascular field, serum circulating miRNAs have proven useful as novel and non-invasive biomarkers in the prognosis and risk assessment of patients with acute coronary syndrome^[173]^. Our group showed that circulating miRNAs can predict disease progression and outcomes in patients with hypertrophic cardiomyopathy and pediatric heart failure^[174]^.

Plasma levels of miR-195-3p are upregulated in both patients with ischemic and non-ischemic heart failure^[175]^. Serum circulating levels of miRNAs miR-499, miR-133a, and miR-208a are elevated and also found to be associated with increasing levels of troponin in patients with acute coronary syndrome^[176 177]^, and patients diagnosed with acute myocardial infarction can also present with higher plasma levels of miR-208b, miR-133a and miR-1^[178]^. In addition, plasma levels of miR-208b and miR-133a are significantly associated with risk of death in patients with myocardial infarction^[178]^. Furthermore, according to the HUNT study, a combination of 5 miRNAs (miR-106a-5p, miR-424-5p, let-7g-5p, miR-144-3p and miR-660-5p) can be a useful addition to the Framingham Risk Score to enhance the risk prediction in acute myocardial infarction^[179]^. Differential expression of specific miRNAs in various disease states make miRNAs a useful and unique tool in heart failure. In spite of the numerous studies that have explored the usefulness of miRNAs in the diagnosis, risk stratification and prognosis of heart diseases, its usefulness seems limited in practicality. Importantly, extracellular miRNAs act as signaling molecules for cell-to-cell communication by being secreted into extracellular fluids, transported via vesicles like exosomes or by binding to proteins like Argonautes^[154]^. These studies highlight the importance of circulating miRNAs as biomarkers, but also as potential factors in contributing to disease progression or in promoting homeostasis.

### miRNAs exert protective effects on the heart

Some miRNAs have also been found to exert protective effects on the heart. Certain miRNAs can protect the heart from various forms of myocardial injury, such as ischemia/reperfusion injury, hypertrophy, and heart failure^[180]^. miR-150 is downregulated and significantly associated with the outcome and severity of heart failure^[181]^. miR-150 exerts its protective effects on the heart by inhibiting proapoptotic Sprr1a^[181]^. miRNAs also play a role in protection against ischemic/reperfusion injury in heart disease. miR-21 has been shown to be cardioprotective against hypoxia/reoxygenation-induced myocardial stress and inflammatory response by inhibiting apoptosis and reducing oxidative stress in cardiomyocytes^[182]^. Chemically synthesized and exogenously administered miR-21 was shown to reduce the size of myocardial infarcts^[183]^. In addition to miR-21, miR-1 and miR-24 exert cardio protection following ischemic/reperfusion injury and improve ischemic tolerance^[183]^. miR-126 has been shown to protect the heart by promoting angiogenesis and reducing myocardial infarction size by targeting hypoxia inducible factor 1 subunit alpha (HIF-1α)^[184]^. The downregulation of miR-133a has been associated with cardiac remodeling, hypertrophy and heart failure suggesting that miR-133a might exert some protective effects on the heart^[185]^. A limitation to these studies is the effect of miRNAs on the regulation of multiple targets, which can result in secondary and undesired effects. More studies are needed to improve specificity of miRNAs in regulating disease processes.

## THE SECRETOME IN CARDIOVASCULAR AGING

Aging is the strongest risk factor for chronic diseases such as heart failure and death^[186]^. With the advent of modern technologies in health, the average life expectancies of people especially in developed world is on the rise^[187]^. There is, hence, an emerging interest on the impact and influence of healthy aging on cardiac function^[188]^. The heart is one of the main organs that is mostly affected in senescence-related diseases. In recent times, there has been a rising interest in the role of the secretome in cardiac aging. The secretome plays a key role in regulating, contributing to and in some cases protecting against age-related diseases in the heart. Senescent cells secrete proteins and miRNAs that can influence cardiac function^[189,190]^. Senescence-associated secretory phenotype (SASP) proteins include GDFs, natriuretic peptides and cytokines, and are positively associated with increasing age^[189]^. GDFs are known as anti-ageing factors whose levels are decreased with increasing age^[59]^. GDFs protect against ischemia/reperfusion injury and promote cardiac regeneration^[59]^. Increasing circulating natriuretic peptides are associated with normal aging, which is a likely result of age-related detriment in cardiac function^[188]^. Senescent cells secrete cytokines that recruit proinflammatory factors in the myocardium, which is related to systemic inflammatory processes observed in response to aging. Understanding protective and detrimental secreted factors is important to target negative consequences of aging.

## THE ROLE OF THE SECRETOME IN PEDIATRIC HEART FAILURE

Heart transplantation remains the best treatment option for children with end-stage heart failure^[191]^. Congenital heart disease, cardiomyopathies and coronary artery disease remain the most common indications for heart transplant in children^[191]^. Amongst all solid organ transplants, heart failure in children has the highest waiting list mortality^[192]^. The long waitlist coupled with post-transplant complications such as graft rejection, failure and infections continue to be major challenges in the management of end-stage heart failure^[193]^. It is possible that the secretome can aid in reducing the risk of acute rejection and/or predict who will respond best to a heart transplant. In addition, there remains a proportion of children with dilated cardiomyopathy that recover from the disease^[194]^. The secretome might be helpful in identifying specific sub-groups of pediatric heart failure patients that may benefit from heart failure transplants or might be better clinically managed until functional recovery^[192]^. This might reduce the rate of unnecessary transplants and associated acute rejection and other post-transplant complications. Our studies have highlighted the importance of circulating factors in pediatric heart failure due to dilated cardiomyopathy^[13]^ and single ventricle heart disease^[195]^, underscoring the importance of investigating the role of circulating miRNAs and proteins as biomarkers and effectors of disease in this underserved and vulnerable population.

## CONCLUSION AND FUTURE AREAS OF RESEARCH

The prevalence of cardiovascular diseases, particularly heart failure, is increasing at an alarming rate, especially in developing countries. The development of novel therapeutic targets and biomarkers can aid in reducing the burden, morbidity and mortality associated with heart failure. The secretome has diverse functions and may exert protective or detrimental effects on the heart. A better understanding of the functional role of these factors can provide more insight into the pathogenesis of heart failure. Secreted proteins and miRNAs have interesting clinical applications in heart failure because minimally invasive techniques are necessary for their detection. The secretome can also prove useful in disease detection and risk stratification. The secreome offers exciting new opportunities for developing sensitive biomarkers, perhaps even better than the traditional markers already in use for diagnosing and treating heart failure. These novel biomarkers can also be integrated into existing scoring systems for heart disease to further improve on their sensitivities and specificities. Evidence for clinical implementation is still lacking and with appropriate investment and research, meaningful headway can be made into improving the diagnosis and treatment of heart failure. The secretome may play an important role in understanding disease causation, progression, diagnosis and prognosis. The advancement of techniques that allow better characterization of the secretome will be of key importance in the translation of research from bench to the bedside.

## Figures and Tables

**Table 1.** Depiction of secreted factors and their roles in disease. Only secreted factors with functional significance are include

Secreted factors	Mechanism of action	Changes in heart disease	Effects in the heart	Levels in heart disease	Potential therapeutic benefits
Natriuretic peptides	Increases sodium loss, inhibits the renin-angiotensin-aldosterone system, decreases vascular pressure, and promotes angiogenesis^[37]^	Plasma concentrations are increased in heart failure^[29]^	Reduces fibrosis of the ventricles^[31,34]^	In heart failure patients, there is a relatively lower level of mature natriuretic peptides but higher levels of the pre-prohormone^[34]^	Can be used as a biomarker and therapeutic target in heart failure, coronary artery disease and myocardial infarction^[29]^
Fibroblast growth factor 21	involved in the regulation of glucose and lipid metabolism^[39]^	This is released in response to myocardial injury^[46]^	Cardioprotective effects by reversing maladaptive hypertrophy^[44]^	Relatively high baseline levels of FGF21 are associated with adverse outcomes in patients with acute heart failure^[46]^	can aid in risk prediction and determining the prognosis^[47]^
Growth differentiation factor	Plays important roles in cell differentiation, growth, and development and inflammation^[51]^	Release is induced by cardiac injury^[55]^	Protect against ischemia/reperfusion injury, promote cardiac regeneration by enhancing cardiomyocyte proliferation and reducing fibrosis in the injured heart^[55,58]^	It is an anti-ageing factor and levels are decreased with increasing age such as age-related cardiomyopathy^[57]^	Can be explored as a novel biomarker in the diagnosis of various heart conditions such as acute coronary syndrome^[52]^
C reactive protein	An acute phase reactant^[66]^	Found in higher concentrations in the acute phase of an inflammatory cascade such as in myocardial infarction^[71]^	Promotes atherogenesis and plays a key role in vascular remodeling^[70]^	Higher levels of CRP are associated with inflammation^[66]^	CRP is a biomarker and a predictor of adverse outcomes in vasculopathy and cardiomyopathy^[72]^
High mobility group box 1	Involved in myocardial necrosis and inflammation^[79]^	Higher levels are associated with cardiac myopathies^[81]^	Play a vital role in cardiac remodeling^[82,85]^	Elevated circulating HMGB1 is associated with myocardial infarction^[85]^	Might be a key therapeutic target in heart failure management
Secreted frizzled-related proteins	Plays a role in cellular differentiation, proliferation, death and early embryonic development^[91]^	Increased serum circulating levels are associated with heart failure^[92]^	sFRPs may promote inflammation, collagen deposition, and fibrosis in healing cardiac tissue^[91]^	sFRP-1 overexpression is associated with an increased risk of cardiovascular diseases^[45]^	May potentially be explored as a biomarker of heart failure
Midkine	It is vital in growth, differentiation and repair ^[105]^	serum levels of MDK are increased in patients with heart failure^[110]^	Reduces the size of an infarct and improve systolic/diastolic left ventricular dysfunction^[113]^	Increased serum MDK levels are associated with the risk factors of atherosclerosis^[111]^	Might play a role as a biomarker of heart failure^[107]^
Endothelin-1	Secreted in response to various stimuli by vascular endothelial cells^[114]^	Associated with cardiac dysfunction^[116]^	Promotes hypertrophic remodeling of cardiomyocytes^[116]^	Induces hypertrophic gene expression ^[116]^	Might be useful as a predictor of adverse outcome in heart failure^[115]^
Sialic acid binding immunoglobulin like lectin 9	Mediates immune signaling in both the innate immune and adaptive immune system^[119]^	Upregulated in the inflammatory cascade^[119,122]^	Involved in cellular apoptosis and neutrophil phagocytosis^[122]^	Elevated in patients with various forms of cardiovascular diseases such as coronary artery disease^[122,130]^	Can be explored in the development of therapeutics for heart failure
Non-metastatic protein clone 23	Maintains the cellular equilibrium of nucleoside triphosphate and regulates the cAMP pathway^[122,126]^	Circulating NM23 suppresses cAMP synthesis in patients with heart failure^[129]^	Causes cardiac hypertrophy^[130]^	Induces expression of hypertrophic genes^[130]^	Elevated levels of circulating NM23 have been found in the sarcolemmal membranes of patients with end-stage heart failure^[130]^
Thrombospondins	Extracellular matrix support and maintains structural integrity of cardiomyocytes^[136]^	Upregulated in pathological remodeling of the heart^[131,134]^	Thrombospondins increase cardiac hypertrophy by increasing extracellular matrix deposition and promoting fibrosis^[132]^	Increased expression is associated with early development of early heart failure and myocardial infarction^[135]^	Potentially useful as a biomarker of cardiac fibrosis^[137]^
Cytokines	Aid in cellular interactions and communication^[142,143]^	Recruits inflammatory mediators to site of cardiac injury^[148]^	Induces cardiomyocyte apoptosis, increases extracellular matrix degradation, increases oxidative stress and DNA damage^[148]^	Elevated and sustained circulatory levels of some cytokines are associated with heart failure^[148]^	Modulation of the inflammatory response in heart failure represent a key potential therapeutic target for intervention^[151]^
microRNAs	Regulate the expression of a large number of genes that play vital roles in cellular processes such as cell growth, differentiation and apoptosis^[155]^	Vital in cardiac biogenesis^[158]^	Plays a role in development and progression of cardiomyopathies and can impact cardiomyocyte remodeling^[157]^	Over-expression or down-regulation of miRNAs have an important impact in cardiac homeostasis and can promote pathological remodeling in animal models ^[164]^	miRNAs have also been investigated as potential therapeutic targets in the management of heart failure^[174]^
